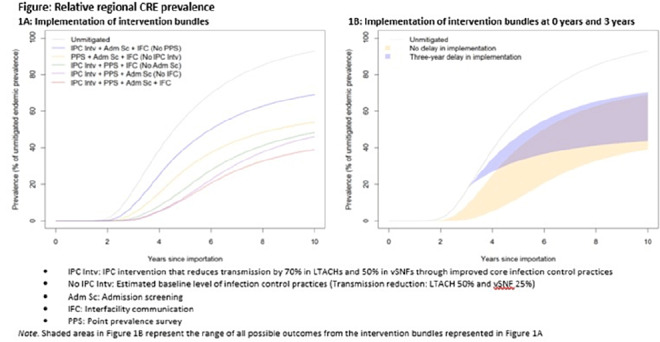# Predicting the regional impact of interventions to prevent and contain multidrug-resistant organisms

**DOI:** 10.1017/ash.2022.78

**Published:** 2022-05-16

**Authors:** Samuel Cincotta, Elizabeth Soda, Rachel Slayton, David Ham, Maroya Walters, Prabasaj Paul

## Abstract

**Background:** Multidrug-resistant organisms (MDROs), such as carbapenem-resistant Enterobacterales (CRE), can spread rapidly in a region. Facilities that care for high-acuity patients with long average lengths of stay (eg, long-term acute-care hospitals or LTACHs and ventilator-capable skilled nursing facilities or vSNFs) may amplify this spread. We assessed the impact of interventions on CRE spread within a region individually, bundled, and implemented at different facility types. **Methods:** We developed a deterministic compartmental model, parametrized using CRE data reported to the NHSN and patient transfer data from the CMS specific to a US state. The model includes the community and the healthcare facilities within the state. Individuals may be either susceptible or infected and infectious. Infected patients determined to have CRE through admission screening or point-prevalence surveys at a facility are placed in a state of lower transmissibility if enhanced infection prevention and control (IPC) practices are in place. **Results:** Intervention bundles that included periodic point-prevalence surveys and enhanced IPC at high-acuity postacute-care facilities had the greatest impact on regional prevalence 10 years into an outbreak; the benefits of including admission screening and improved interfacility communication were more modest (Fig. 1A). Delaying interventions by 3 years is predicted to result in smaller reductions in prevalence (Fig. 1B). Increasing the frequency of point-prevalence surveys from biannually to quarterly resulted in a substantial relative reduction in prevalence (from 25% to 44%) if conducted from the start of an outbreak. IPC improvements in vSNFs resulted in greater relative reductions than in LTACHs. Admission screening at LTACHs and vSNFs was predicted to have a greater impact on prevalence if in place prior to CRE introduction (~20% reduction), and the impact decreased by approximately half if implementation was delayed until 3 years after CRE introduction. In contrast, the effect of admission screening in ACH was less (~10% reduction in prevalence) and did not change with implementation delays. **Conclusions:** Our model suggests that interventions that limit unrecognized MDRO introduction to, or dispersal from, LTACHs and vSNFs through screening are predicted to slow distribution regionally. Interventions to detect colonization and improve IPC practices within LTACHs and vSNFs may substantially reduce the regional burden. Prevention strategies are predicted to have the greatest impact when interventions are bundled and implemented before an MDRO is identified in a region, but reduction in overall prevalence is still possible if implemented after initial MDRO spread.

**Funding:** None

**Disclosures:** None

**Figure:**